# Patient experiences of being advised by a healthcare professional to get pregnant to manage or treat endometriosis: a cross-sectional study

**DOI:** 10.1186/s12905-023-02794-2

**Published:** 2023-11-30

**Authors:** Diksha Sirohi, Sylvia Freedman, Lesley Freedman, Gretchen Carrigan, Alison J. Hey-Cunningham, M. Louise Hull, Rebecca O’Hara

**Affiliations:** 1 Robinson Research Institute, Adelaide Medical School, University of Adelaide, Ground Floor, 55 King William Road, North Adelaide, SA Australia; 2 EndoActive, 16 Pashley St, Balmain, Sydney, NSW Australia; 3 Central Clinical School, Faculty of Medicine and Health, The University of Sydney, Camperdown, NSW Australia

**Keywords:** Endometriosis, Pregnancy, Healthcare professionals, Fertility

## Abstract

**Background:**

There is a lack of evidence that pregnancy reduces endometriotic lesions or symptoms, however studies indicate that people with endometriosis are commonly advised to get pregnant to manage or treat endometriosis. This study sought to examine the impact of this advice on patients with endometriosis when the advice was provided by healthcare professionals.

**Methods:**

The Endometriosis Patient Experience Survey was a self-reported, community-based, cross-sectional online survey of people who had been medically diagnosed with endometriosis. Descriptive statistics were used to analyse the quantitative survey data and thematic analysis was undertaken for the qualitative survey data.

**Results:**

1892 participants had received the advice to get pregnant or have a baby to manage or treat their endometriosis, with 89.4% of participants receiving this advice from healthcare professionals. In exploring the qualitative data, seven themes were contextualised relating to the impact of this advice in terms of health literacy, accepting the advice, rejecting the advice, major life decisions, healthcare interactions, mental health and relationships.

**Conclusions:**

This study demonstrates profound and often negative patient impacts of the advice from healthcare professionals to get pregnant to manage or treat endometriosis. Impacts ranged from planning for pregnancy, hastening the making of major life decisions, eroding trust with healthcare professionals, worsening mental health and straining relationships. Providing evidence-based information on the treatment and management of endometriosis is essential. Pregnancy or having a baby should not be suggested as a treatment for endometriosis and the provision of this advice by healthcare professionals can have negative impacts on those who receive it.

## Background

Endometriosis is a chronic condition, affecting approximately 1 in 9 women and those assigned female at birth by age 44 [1]. Symptoms vary but the condition is typically associated with dysmenorrhoea, chronic pelvic pain, dyspareunia and bowel disturbances [2]. Diagnosis is often delayed with an average delay of 6.4 years [3, 4]. Lived experiences of people with endometriosis reveal that symptoms are often dismissed as normal by healthcare professionals and the wider community [5]. The delay in diagnosis can lead to chronic pelvic pain, fatigue, anxiety and depression [3] negatively impacting the patient’s quality of life [4].

Subfertility /infertility is one of the symptoms commonly associated with endometriosis [4, 6, 7]. The incidence of infertility is 30–50% higher for people with endometriosis [8], although many people with endometriosis can conceive without fertility treatment. There is also a 25–50% higher incidence of endometriosis in infertility populations [9]. There are many endometriosis-related causes of difficulty conceiving including painful sex at ovulation [10], disrupted function of the fallopian tubes due to adhesions [11], lower numbers of eggs to find one with pregnancy potential [12] and the lining of the womb being less responsive to progesterone [13]. Overarching this is the cyclic response of endometriosis to reproductive hormones which leads to cells that shed in ectopic lesions during menstruation being unable to leave the body, like the endometrium that lines the womb [14]. The immune system is thus required to launch an inflammatory, innate immune response to phagocytose dying endometrial cells in the pelvis [15]. This hostile environment makes it more difficult for eggs, sperm [16] and embryos [17] to function and conception can be delayed. Some evidence suggests that endometriosis can develop during pregnancy [18]. Signorile et al. reported the presence of endometriosis in a 25-week-old female fetus with a proposed mechanism that it may be caused due to the dislocation of primitive endometrial tissue outside the uterine cavity during organogenesis [18]. Research on fertility and endometriosis focuses on preserving or improving fertility to maximise the chance of conception. However, fertility-related conversations are known to be sensitive [19] and may not be a priority for those seeking medical help for endometriosis [20].

Additionally, misinformation on endometriosis treatment has been reported in the literature and is perpetuated in practice, among lay people, and online [21, 22]. One of these fallacies is that pregnancy is a cure for endometriosis [23]. Similar findings have been reported in other studies [20, 24–27], showcasing that receiving this advice is common and pervasive within the community. However, a recent systematic review indicates that there is no evidence that pregnancy can reduce the size or number of endometriotic lesions and concludes that evidence for an association between pregnancy and endometriosis symptoms is controversial and strongly biased [22]. The European Society for Human Reproduction and Embryology (ESHRE) Guidelines for Management of Endometriosis state that pregnancy should not be recommended as a cure for endometriosis, as it does not always lead to improvement in symptoms or arrest disease progression [28]. Despite this, pregnancy is often recommended as a treatment strategy in practice [20, 22, 24–27]. There is a paucity of research that examines how people with endometriosis receive the advice to get pregnant to manage or treat endometriosis symptoms and how it impacts their lives. This paper explores the lived experiences of patients who have received this advice from healthcare professionals.

## Methods

The Endometriosis Patient Experience Survey (EPES) was a collaboration between EndoActive [29] an Australian endometriosis awareness, information and advocacy organisation, The University of Sydney and the Robinson Research Institute, University of Adelaide. Ethics approval was obtained from The University of Sydney Human Research Ethics Committee (Protocol number 2017/155). It was a self-reported, community-based, cross-sectional online survey of people who were 18+, had been medically diagnosed with endometriosis (either through surgery or through a medical diagnosis from a healthcare professional), and who could read and understand English. The participants were recruited through EndoActive [29], other endometriosis patient groups, community groups, social media posts, digital media organisations, fertility clinics, pain associations and research centres. The online survey was hosted on Survey Monkey. Data were collected from May 2017 to October 2017.

The survey consisted of 65 items and included a mixture of fixed-response and open-ended questions about demographics, diagnosis, surgical history, and experiences of being provided different advice concerning endometriosis (e.g. pregnancy, hysterectomy, management strategies) and outcomes of this advice. The survey was designed by the research team and refined by an expert panel review of endometriosis clinicians, nurses, researchers and endometriosis patient group representatives. Quantitative data were downloaded from Survey Monkey and imported into SPSS (v.26) for analysis. Frequencies for categorical data and mean, SD and 95% confidence interval (CI) of the mean for continuous data are reported. Where multiple responses are accepted in the question an independent analysis of each response option was conducted with the numerator the number of people with this response over the denominator which is the number of people that answered the question.

NVIVO 12 qualitative analysis software (QSR International, Massachusetts USA) was used to store and organise the qualitative data. Thematic analysis was manually conducted using Braun and Clarke’s approach [28]. The six-step method (familiarisation with the data, generating initial codes, identifying themes, reviewing themes, defining the themes and final writing) was used to analyse and describe patterns across the dataset that were then combined into broader themes [30]. One researcher (DS) read the transcripts multiple times to generate an initial set of codes. Codes were then grouped into sub-themes and themes. Another researcher (RO) cross-checked the coding. DS and RO re-grouped the codes as required after discussion and mutual agreement. This paper presents the analysis of the open-ended question “Can you tell us how the advice to consider getting pregnant or having a baby impacted your life in the next 12 months?”.

## Results

A total of 3347 eligible participants completed the survey. Of the 3347 participants, 1892 had received the advice to get pregnant or have a baby to manage or treat their endometriosis. The characteristics of these participants are summarised in Table 1. Over two-thirds of these participants resided in Australia and the majority of participants reported that their endometriosis was diagnosed through surgery. The majority of participants reported 1–3 surgeries for endometriosis. Permission to determine the revised American Society for Reproductive Medicine (rASRM) [31] stage of endometriosis via operation notes or medical reports were not available for this study and thus are not reported.

**Table 1:** Summary of participant characteristics

Characteristic	Value
Age in years (Mean [95% CI])	33.4 (33.0–33.7)
Residing in Australia (**% [n])**	64.5% (1221)
Endometriosis diagnosed by surgery^a^ (**% [n])**	92.2% (1744)
Number of laparoscopies or laparotomies for endometriosis^b^ (**% [n])**	
1	34.9% (608)
2	27.1% (473)
3	14.9% (259)
4+	23.1% (404)

^a^The remainder of participants were medically diagnosed (e.g. told by a doctor they had endometriosis)

^b^Among those surgically diagnosed

The majority of participants who had been recommended to get pregnant or have a baby to manage or treat their endometriosis indicated that a healthcare professional had provided this advice (89.4%, n = 1691). This was followed by advice from a family member (24.5%, n = 464), friend (22.0%, n = 417), acquaintance with endometriosis (15.7%, n = 297), can’t remember (2.0%, n = 37) and other (1.9%, n = 36; multiple responses allowed).

Participants who indicated that they were advised by a healthcare professional (n = 1691) received the recommendation from gynaecologists (72.0%, n = 1218) and GPs (45.5%, n = 769; multiple responses allowed). At the time of receiving this advice, participants were between 12 and 45 years of age (mean age: 25.1, 95% CI 24.9–25.4) but only 20.4% (n = 339) had asked their healthcare professional for advice on getting pregnant or having a baby.

Patients reported three main reasons healthcare professionals mentioned for providing the advice to get pregnant or have a baby (multiple responses allowed):

1. As a treatment for endometriosis (68.2%, n = 1153).
2. Because endometriosis can affect fertility (56.1%, n = 948).
3. As a cure for endometriosis (36.8%, n = 622).

‘Treatment’ refers to the ability to moderate disease symptoms such as pain and was the most common reason for pregnancy advocacy. The concern that a window of fertility could be lost if conception was left too late was the next common reason. Pregnancy was also commonly presented as a cure for endometriosis.

Participants indicated that at the time the advice was provided, it was presented as something they should do immediately 36.9% (n = 616), within the next two years (21.2%, n = 354), to consider in five years (8.7%, n = 146) or to consider in the future when they were ready (22.8%, n = 380); with 10.4% (n = 173) responding that they were unsure of the advised timeframe.

The personal circumstances of participants at the time that this advice was received from healthcare professionals are summarised in Table 2. Whilst the majority of participants were in a relationship at the time of the advice, less than 20% reported being interested in having a baby at the time. Further, almost half of the participants reported feeling too young to have a baby. Overall, only a third of respondents who received this advice felt that it was appropriate given their situation at the time.

**Table 2:** Circumstances of participants (n = 1691) who received pregnancy advice from a healthcare professional

Personal circumstances at the time the advice was provided	
In a relationship	72.2% (n = 1198)
With a person they wanted to have a baby with	46.1% (n = 766)
Interested in having a baby at the time advice was given	18.1% (n = 301)
Felt well enough to consider having a baby	19.8% (n = 329)
Felt could financially support a baby	27.8% (n = 461)
Felt too young to have a baby	42.2% (n = 700)

The majority of these participants reported that their healthcare professional had enquired or was aware of their personal circumstances (62.5% [n = 1037]). However, less than half of these participants reported that their healthcare professional had enquired about their interest in having a baby (44.3% [n = 736]). A third reported that the healthcare professional was aware of or had discussed age before providing the advice (34.9% [n = 579]).

## Thematic analysis

**Fig. 1:**
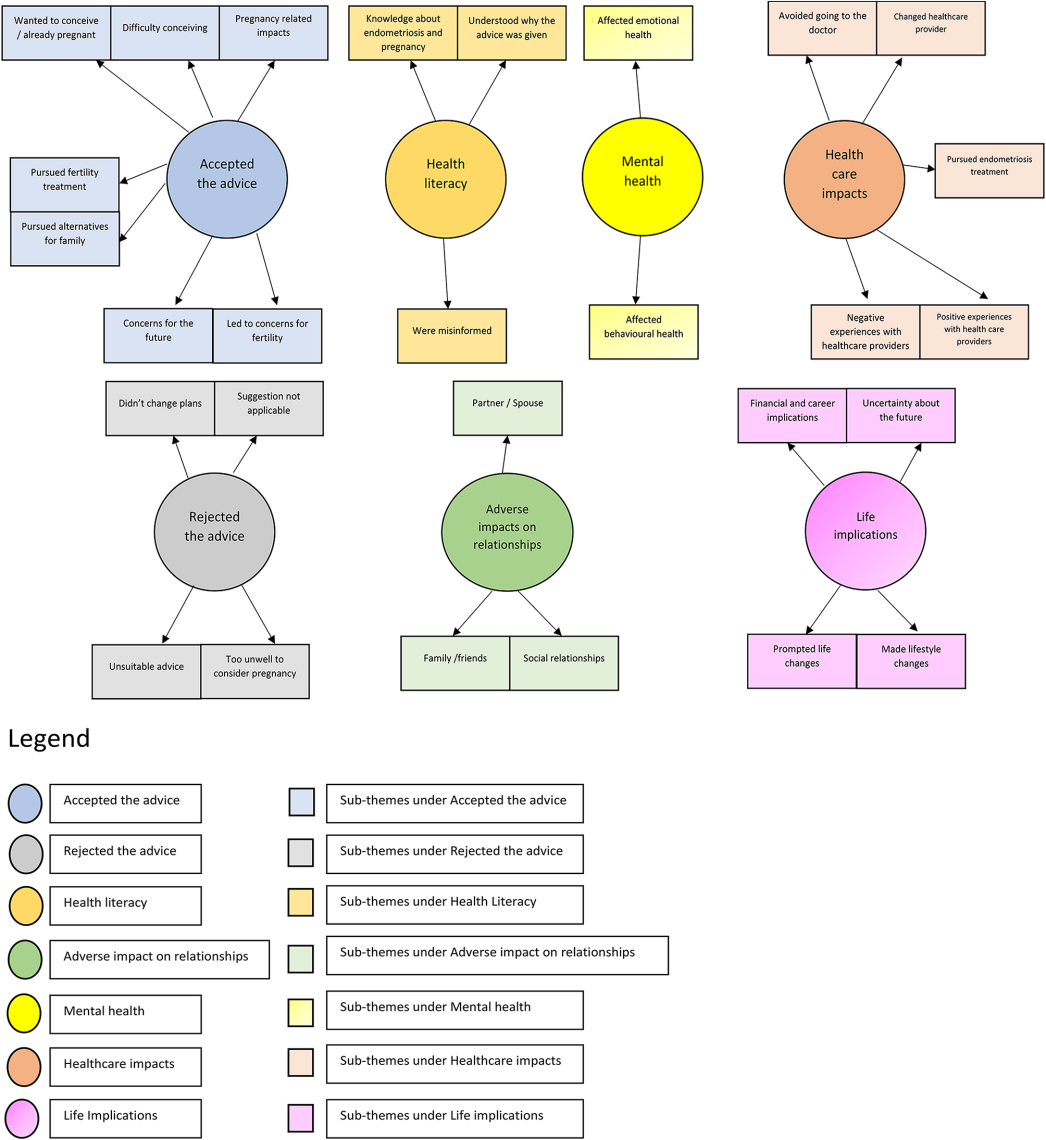
Thematic analysis map

Among those who were advised by a healthcare professional to get pregnant to manage or treat endometriosis, a total of 1570 participants responded to the open-ended survey question “How did the advice to consider getting pregnant or having a baby impact your life in the next 12 months?”. Seven major themes were identified in the responses to this question (see Fig. 1).

### Theme 1 - Health Literacy: Misinformation that pregnancy cures endometriosis

Some participants’ responses indicated that the advice to consider getting pregnant had an impact on their knowledge about endometriosis, fertility and pregnancy. After receiving the advice to get pregnant, some participants stated that they felt their knowledge was improved. Other participants stated that they knew pregnancy wasn’t a cure for endometriosis and sought information to refute the advice they were given. They mentioned feeling ‘disappointed’, ‘misinformed’, ‘surprised’, ‘irritated’, ‘angry’, ‘offended’ and ‘frustrated’ on receiving this incorrect advice.

*Didn’t take it [the advice] on board as I’ve read numerous times it’s [pregnancy is] not proven as a treatment [for endometriosis].*

Some respondents acknowledged that the advice to get pregnant was given to address fertility concerns, but they were not seeking fertility advice at the time. However, other participants did believe pregnancy would alleviate their symptoms or felt they should have received advice to consider having a baby earlier.

*Just that I knew I would probably have a hard time falling pregnant, but it may give me some relief for a few years.*

### Theme 2 - Accepted the advice: The ‘pressure’ and ‘obsession’ for a baby

Many participants indicated that their health professional’s advice to conceive, prompted them to try for conception or pursue treatments such as surgery to facilitate conception. Participants also reported using artificial reproductive techniques [30] such as in-vitro fertilisation (IVF) and freezing eggs for future pregnancy. Some participants described feeling stressed or pressured for a baby and rushed into considering pregnancy.

*At the moment we are trying to seek fertility treatment as we have been unsuccessful for the past 2 years in trying to conceive.*

Participants reported concern about their ‘ability to have children in the future’. Some indicated that they were ‘unsure whether they wanted children’ and yet others felt that they had ‘no choice’ but ‘to consider having a baby’. Some participants had adopted a baby or used surrogacy to complete their family. A few even described becoming ‘obsessed’ with the idea of having a baby.

*I became obsessed with babies, pregnant women, getting pregnant, the thought that I would never be able to give birth to my own children.*

A small number of participants reported being happy with the advice. Among participants who did have a baby, post-partum issues such as ‘worsening of endo symptoms’ and ‘not receiving post-partum care for endometriosis management’ were raised as a concern.

### Theme 3 – Rejected the advice: ‘Inappropriate’ and ‘unsuitable’

Some participants described the advice to have a baby as unsuitable or inappropriate for their situation and rejected it. They reported ‘not [being] in a position to have children’ or ‘not ready for a baby’ or were ‘too young’ to have children. People indicated being single or not being in a relationship in which they wanted a baby.

*Didn’t take it [the advice] on board.*

*I was 13. It wasn’t appropriate.*

*I ignored it [the advice to get pregnant] and continued with my high school studies.*

Others were still at university with no financial independence and felt that the advice to have a baby was inappropriate for their circumstances.

*Given that I was 19 at the time, I knew that in the future [I] wanted to be a mother but [I] knew that it wasn’t at that stage in my life yet.*

*I was 21, single and at uni so it simply wasn’t an option.*

Yet others disregarded the advice as they already had children, felt they were ‘getting too old’ to have children or had decided not to have children at all. Some respondents mentioned ‘being too unwell’ to have a baby.

*As bad as I felt I knew in my heart I wouldn’t be well enough to take care of a baby. I could barely walk [two and a half] weeks out of the month*.

A small number of participants ‘regretted’ not following the advice, stating that they ‘made the wrong choice’.

### Theme 4 - Life implications: The impact on career, finances and major life decisions

Participants reported that advice to become pregnant or have a baby impacted their lifestyle, finances, career and major life events. They described how the advice to have children ‘prompted major life changes’, including searching for a suitable partner, marrying earlier than planned, moving country, considering having children earlier than planned, considering IVF treatment sooner rather than later, and putting off major expenses such as buying a house.

*Brought forward plans to have a baby by several years, despite reservations of my fiancé. I felt quite stressed and that it was a race against time to conceive.*

*I rushed my plans over the next 5 years.*

Some participants reported stressful financial impacts, including affordability issues concerning ART procedures such as IVF.

*After 6 months of trying, I couldn’t get pregnant naturally, so we started IVF. First IVF resulted in [an] ectopic pregnancy which was very painful. 2 years later and after another 6 stimulated cycles of IVF, the endo grew back into my bowel requiring another large bowel resection and removal of other organs. Another 2 stimulated IVF cycles after that. So, 9 IVF rounds in total, we are broke financially, emotionally and physically.*

Participants described ‘needing to take time off work’ as affecting their career and that planning a family required them to re-evaluate their career.

### Theme 5 - Healthcare impacts: Experiences with the healthcare system

Participants reported that the advice they were given impacted their other experiences with the healthcare system. They mentioned delayed diagnosis, unmet needs, and feelings about interacting with healthcare professionals. Participants reported a mismatch between what they wanted (e.g., pain management) and the advice they were provided.

*The pain and other issues I was experiencing weren’t dealt with to an adequate level.*

*The fact that I wasn’t given any advice suitable to me was very upsetting. I travelled & spent a lot of [dollars] trying to find a practitioner to help with pain management.*

Others reported that prescribed treatments didn’t work well for them (e.g., hormonal treatment). A few indicated seeking ‘a second opinion’ as they were ‘dissatisfied’ with the advice to get pregnant. Others turned to ‘alternative medicine’ to manage their condition. Many participants expressed that such experiences led them to ‘lose faith’ in the medical profession while others mentioned that they ‘avoided going to the doctor’ or ‘changed their healthcare professional’.

*I ignored it [the advice to get pregnant] and continued with my high school studies. But it likely led to me actively avoiding having to visit that GP.*

Some participants reflected on a delay in diagnosis or being diagnosed with other conditions before receiving their endometriosis diagnosis.

*The first time [I] complained about the pain in my mid-twenties, [I] was told it was just my body and no one checked to see if [I] had endo, so I lost a tube, and the disease decimated my egg reserve, not enough information.*

Some participants reported having a positive experience with healthcare professionals around receiving the advice. They were looking for ‘fertility advice’, were satisfied that ‘fertility issues were addressed’ and reported that ‘their doctor was supportive’.

*Fertility issues and endometriosis were diagnosed and treated, and I had a baby. Obviously, that advice isn’t going to be right for everyone. In my circumstances wanting to get pregnant led me to finding out I had endometriosis.*

### Theme 6 - Mental health: The emotional and behavioural aspects

Many responses touched on the theme of mental health impacts of the advice to get pregnant and have a baby. Participants reported the advice had impacts such as being ‘anxious’, ‘panicked’, ‘stressed’ and/or ‘depressed’. Some people required help from a psychologist.

*It [having a baby] was in my mind every day and added a significant amount of stress to day-to-day life. It is a huge decision to make, endometriosis or not. I was very confused and depressed.*

*Depressed at the prospect [I] may never have children.*

*It was a nightmare for me and something that impacted me greatly on an emotional level.*

*Frustration. Feeling invisible. Feeling irrelevant. Angry. Disappointed. Deflated. Seen as a baby making oven instead of being seen as a person.*

Additionally, participants described feelings of ‘isolation’, ‘dismissal’, and ‘loneliness’ about being given this advice. Respondents reported having negative self-beliefs such as feeling ‘powerless’, ‘low self-esteem’ and feeling ‘un-womanly’ when their fertility was affected due to endometriosis.

### Theme 7 – Adverse impact on relationships

The advice participants received impacted partner/spouse relations, family, friends and social relationships. Those with partners reported that discussing the pregnancy advice with their partner was stressful and couples felt ‘pressured’ to have a baby.

*I talked with my husband but decided we didn’t want to be pressured into having a child when we weren’t ready just because of endometriosis.*

Some participants also reported that their relationship was adversely impacted by the advice due to differences in partner desires or readiness to have a child.

*It ended up ruining my relationship as I felt a huge pressure to have kids young and my partner couldn’t understand the intense conversation at a young age.*

Some participants also described negative impacts on their sexual relationships. They stated that they experienced painful sex due to endometriosis. Given the pain, the rush for a baby added further stress.

*I can’t really have sex because of the pain. [My partner] knew this, but still thought I should have a kid. This triggered depression around my sexual dysfunction and inability to be in a normal sexual relationship.*

Participants who were not in relationships in which they wanted to have a child also described strain.

*I was in an abusive relationship. The nurse said it in front of my partner who then decided I no longer needed OCP. I fell pregnant on the first cycle but luckily for my own safety found out 2 weeks after leaving him. Terminated pregnancy.*

Participants indicated that they also experienced challenges with other relationships, feeling ‘as if family members didn’t understand their struggles’. Some said that they experienced family pressure to find a suitable partner to have a baby with.

*When I told my parents, I could feel their hope that I might find an older man who could “look after me” and with whom I might start a family with. I didn’t feel pressure to find this person, but I could tell that’s what they wanted for me and put that pressure on myself, whilst at the same time thinking that no man would want to be with someone who had so many health problems. I was very lost.*

## Discussion

The EPES study revealed profound and wide-ranging impacts of healthcare professionals’ advice to have a baby for people with endometriosis. Impacts were evident across several domains including health literacy, the timing of pregnancy and life decisions, interactions with health care services, mental health, and relationships. This advice negatively impacted some people by perpetuating misinformation about the management or treatment of endometriosis, contributing to feelings of ‘pressure’ or ‘obsession’ about having a baby, being considered ‘inappropriate’ or ‘unsuitable’, rushing their major life decisions, compromising future healthcare interactions through erosion of trust, being detrimental to mental health, and by adding tension to personal relationships.

In this survey, more than half of the participants (1692 of 3347 total) reported that they had been advised by a healthcare professional to conceive to manage or treat their endometriosis. Our study solely focuses on pregnancy advice in an endometriosis population, although five other studies [20, 24–27], included the impact of pregnancy advice in the management of endometriosis within a broader survey and concur with our findings. An Italian study reported that women with endometriosis often felt pressured to conceive by their healthcare providers even though they were not ready to have a baby, which was associated with anxiety disorders and recurrent panic attacks [32]. One study in North California, USA [33] and one dissertation abstract from the University of Massachusetts Amherst [34] reported that adolescents and young adults felt pressured or rushed to have a baby after receiving an endometriosis diagnosis.

Available evidence does not support pregnancy being beneficial in reducing the symptoms of endometriosis, except in the specific context of continued cessation of menstruation and therefore associated dysmenorrhea [22, 35]. The ESHRE clinical guideline for endometriosis reflects this, expressly stating “Patients should not be advised to become pregnant with the sole purpose of treating endometriosis, as pregnancy does not always lead to improvement of symptoms or reduction of disease progression” [28]. Giving fertility advice, as distinct from pregnancy advice, may be appropriate depending on the age and circumstance of the patient, as fertility can be impacted by endometriosis [6, 7, 36, 37]. For those concerned about fertility, ovarian reserve testing can be offered, and egg and embryo freezing are possible options to preserve future fertility. Thus, if fertility concerns are raised by a person with endometriosis, it should prompt a much more complex discussion than a simple recommendation to conceive promptly.

Like others [20], our study shows that fertility-related conversations are prioritised over other more pressing concerns of people with endometriosis such as pain management, which causes negative interactions with health professionals. This may stem from sociocultural beliefs that women may ‘want to’ or ‘should be mothers’ [38, 39]. Independent of the rationale, suggesting pregnancy as a treatment is neither evidence-based [22, 28] nor welcome among those who are not seeking fertility advice.

Survey participants clearly described advice to get pregnant as inappropriate or unsuitable to their situation or desires. There is an identified need for healthcare professionals to listen to and include patients as partners [40] when formulating an optimal endometriosis healthcare plan [20, 41]. However, often healthcare interactions do not provide adequate time for individualised evaluation and management planning [42]. Our study supports recommendations for patient-centric conversations to devise appropriate endometriosis care plans [43] where patients play an active role in setting up their care plan. This empowers patients in their medical decision-making and results in tailored age-and-situation-appropriate advice and is aligned with the UK’s National Institute for Health and Care Excellence [44] guidelines.

Our study indicated that healthcare professionals’ advice that was not appropriate to the patient’s situation or needs had negative impacts on future healthcare interactions. Participants reported losing faith in healthcare professionals, changing providers, or avoiding, healthcare. Other studies have highlighted the crucial impact healthcare professionals have on endometriosis patients’ experiences of healthcare [45], and the importance of trust and legitimacy to a group that often feels unheard or dismissed by healthcare professionals [46].

One way to avoid this negative impact would be to improve healthcare professionals’ education about endometriosis. There is an identified need to improve doctors’ education about endometriosis [42, 47, 48]. Healthcare professionals themselves report limitations in expertise with respect to endometriosis management and face challenges in providing best-practice care [45, 49]. Designing educational programs aimed at improving doctors’ education on endometriosis treatment modalities is a crucial aspect of improving endometriosis patient experience. Beyond the need for education, there is also a need for models of multi-disciplinary, collaborative care and evaluation through the use of consumer-informed well-being, quality of life and satisfaction measures [49].

There is a need for community and patient education to improve patient experience. Patient education is recognised as one of the goals for improving endometriosis care [42]. A school-based menstrual health program in New Zealand, that provides education to students on endometriosis, led to a shift in the early presentation of young women with endometriosis to specialised endometriosis care centres [50]. Patient and community education can be aided by providing evidence-based educational resources on endometriosis such as the EndoZone digital health platform [51] co-created by the Australian endometriosis community, clinicians and researchers; EndoActive’s suite of educational videos: ‘Shared Perspectives on Endometriosis’ [29], designed for patients and healthcare professionals; Pelvic Pain Foundation of Australia’s online resources for healthcare professionals and consumers [52], including the Pelvic Pain Education Program Talk [53] available as a free educational resource in schools [53], and QENDO’s freely available endometriosis app for consumers [54].

### Strengths and limitations

This study explores various nuanced impacts, as described by endometriosis patients, of being advised by healthcare professionals to get pregnant to manage or treat endometriosis. The strengths of this study are the large sample size and the ‘richness’ of the qualitative information. Furthermore, the study contributes to the growing body of evidence on the need for patient-centric conversations in endometriosis management and education for healthcare professionals and patients alike.

While this study explores in detail an aspect of the endometriosis patient experience of the mostly Australian respondent cohort, it does not necessarily represent all experiences of the endometriosis community, nor is it designed to reflect a clinical perspective. Recall bias is inherent in community-based cross-sectional surveys asking participants to reflect on their experiences [55] and is a weakness of the study. We acknowledge that this survey relies on the self-reported experiences of patients and many issues may be discussed within one consultation such as receiving an endometriosis diagnosis, fertility-related concerns, pain management and the financial cost of treatment, therefore, it is difficult to elicit the true cause of psychological and socioeconomic distress. Lastly, we did not formally assess quality of life using standardised forms.

## Conclusion

More than 50% of participants in the EPES study reported being advised by a healthcare professional to plan a pregnancy to manage their endometriosis, despite little evidence supporting this advice and international clinical guidelines which contradict it. We demonstrate that the advice negatively impacted patients with respect to timing of pregnancy and life decisions, relationships and mental health, health literacy, and interactions with health care services. While the nuances of fertility-related matters affect emotional well-being and alter important life decisions, they are not always a priority for people with endometriosis. Patient-centric approaches and healthcare professionals’ education are crucial for positive patient experiences. Doctor-patient communication is likely to improve if patients are asked about their fertility-related preferences, if their healthcare plans are co-developed with their needs prioritised, and if evidence-based advice is given for the management of endometriosis. Pregnancy or having a baby should not be suggested as a treatment modality for endometriosis.

## Data Availability

The datasets generated and analysed during the current study are not publicly available as participants did not consent for data sharing but are available from the corresponding author upon reasonable request.
